# Cozen’s deformity: resolved by guided growth

**DOI:** 10.1007/s11751-018-0309-y

**Published:** 2018-03-16

**Authors:** Matthew Morin, Joshua Klatt, Peter M. Stevens

**Affiliations:** 1 Department of Orthopedics, University of Utah, Salt Lake City, USA; 2 University of Utah, Salt Lake City, USA

**Keywords:** Cozen’s phenomenon, Tibial valgus, Guided growth

## Abstract

Proximal tibial metaphyseal fractures in children can lead to progressive and symptomatic tibial valgus. Corrective osteotomy has been abandoned, due to frequent complications, including recurrent valgus deformity. While spontaneous remodelling has been reported, this is not predictable. For children with persistent deformities, we have resorted to guided growth of the tibia. We present 19 patients who were successfully treated with guided growth, tethering the proximal medial physis. There were ten boys and nine girls, ranging in age from two to 13.6 years at the time of intervention. The mean follow-up from injury was 7.3 years. We documented the intermalleolar distance, mechanical axis deviation (by zone), medial proximal tibial angle (MPTA), and leg length discrepancy. Removal of the plate, or more recently, the metaphyseal screw, was undertaken upon normalization of the mechanical axis. Including the four patients who have undergone repeat tethering for recurrent valgus (one patient—twice), we are effectively reviewing 24 Cozen’s phenomena, making this the largest series reported in the literature. Correction of the mechanical axis and the proximal medial tibial angle was achieved in all but one patient. Limb length inequality at follow-up ranged from 0.1 to 1.5 cm, with a mean of 0.5 cm. There have been five recurrences in four patients to date; four corrected with repeat tethering and one is pending. Two patients developed significant over correction because of parental failure to pursue timely follow-up. Both have corrected to neutral with lateral tibial physeal tethering. Ten patients have attained skeletal maturity and required no further treatment. The remaining nine patients will be followed until maturity. Guided growth is an excellent choice for the management of post-traumatic tibial valgus. Our rationale for restricting medial overgrowth is twofold: (1) to restore the MPTA and (2) to reduce the length discrepancy due to tibial overgrowth caused by the fracture. Recognizing the potential for recurrent deformity following implant removal, our standard practice now includes removal of just the metaphyseal screw and subsequent reinsertion, in the event of rebound valgus deformity.

*Level of evidence* Therapeutic IV, retrospective series/no control cohort.

## Introduction

Post-traumatic tibial valgus is a recognized phenomenon following proximal metaphyseal fractures in children [1–7]. Less frequent causes include biopsy, bone graft harvesting, tibial traction pin insertion, and infection. Tibial valgus can occur despite optimal initial fracture management, creating a dilemma for the orthopaedic surgeon, faced with persistent or progressive deformity and unhappy parents. One management strategy is to observe the patient rather than intervene, as some authors have shown that mild deformity can correct spontaneously [2, 8, 9]. However, with observation alone, distal tibial compensatory varus deformity can lead to a “serpentine tibia” [10]. The mechanical axis remains lateral to the centre of the knee, eccentrically loading the lateral knee structures and potentially compromising patella-femoral stability. Moreover, there may be long-term problems with the resulting ankle varus [11].

By consensus, proximal tibial osteotomies are contraindicated due to the high rate of complications including neurovascular injury, compartment syndrome, and a high rate of recurrence [4, 12, 13]. If spontaneous correction of the deformity is not observed and there is sufficient growth remaining, hemi-epiphysiodesis can offer the optimal solution [10, 14, 15]. Permanent epiphysiodesis, be it open or percutaneous, can only be used in those approaching skeletal maturity, recognizing that, if timing is incorrect, this can result in over- or under-correction and leg length discrepancy. In contradistinction, reversible physeal tethering (guided growth) can be offered at any age and repeated as needed. We will discuss the results of proximal medial tibial hemi-epiphysiodesis using an extra-periosteal 2-hole plate to mitigate the medial tibial overgrowth due to Cozen’s phenomena.

## Materials and methods

This review received IRB approval for a consecutive series of patients treated at our institution. Our inclusion criteria were as follows: metaphyseal fracture of the proximal tibia (± fibula) followed by post-traumatic valgus deformity. A total of 21 patients were treated with guided growth and met these criteria. Patients’ charts and radiographic data were retrospectively reviewed. We excluded two patients based on incomplete radiographic records or incomplete follow-up. There were 11 males and eight females with the average age of 4 years and 7 months at time of injury. Review of post-treatment films at the time of the initial injury showed that no patients had a malunion at the time of healing. Unfortunately, none of the patients had full-length radiographic views that allowed measurement of their mechanical axis at this point in time.

Long leg casting was the initial fracture treatment of choice in all but one patient who was treated with a plate and screw construct due to an open fracture in unacceptable alignment. None of the initial treatment notes had comments, indicating that clinical examination at the time of cast removal showed any asymmetry of alignment with the contralateral knee. Every case was significant for insidious medial tibial overgrowth, leading to malalignment and which was associated with a circumduction gait pattern. At an average of 27 months post-injury, all patients were treated with guided growth using a single, extra-periosteal 2-hole plate to bracket the proximal medial tibial physis. The surgery was performed on an outpatient basis, with no immobilization or activity restrictions imposed post-operatively. Clinical examination included measurement of limb length inequality and observation of the gait pattern before performing guided growth and at periodic follow-up.

**Fig. 1:**
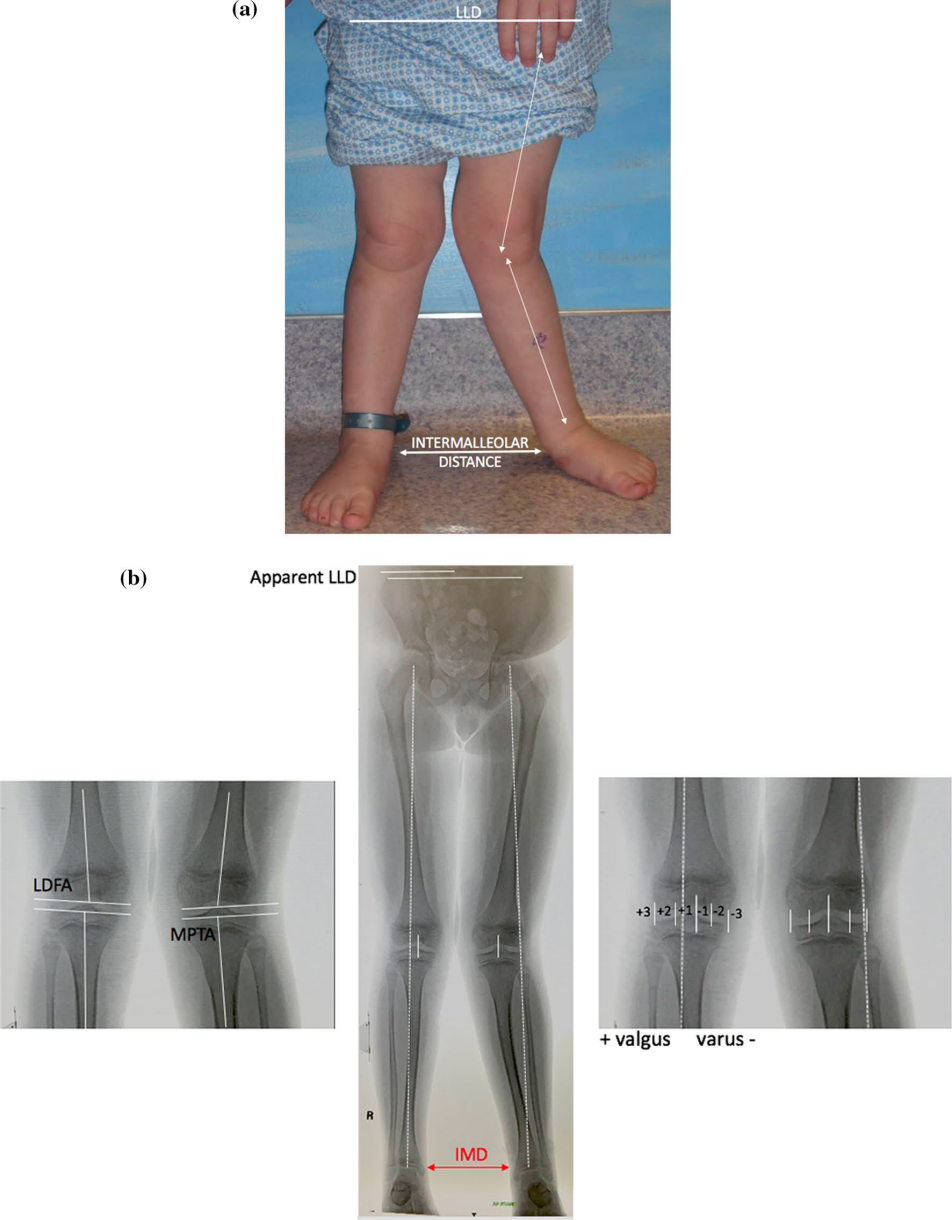
**a** Clinical measurements included the relative limb lengths and the intermalleolar distance (IMD). **b** The radiographic measurements comprised of the mechanical axis zone (by quadrants), the limb lengths, and the anatomic angles, including the lateral distal femoral angle (LDFA) and the medial proximal tibial angle (MPTA)

Full-length standing AP radiographs were obtained pre-operatively and at follow-up, comparing the affected to the uninvolved limb. Radiographic measurements included the mechanical axis deviation and zone (by quadrant), leg length discrepancy, lateral distal femoral angle (LDFA), and medial proximal tibial angle (MPTA) (Fig. 1). Post-operatively, the recommended follow-up was at 3-month intervals until correction of the mechanical axis to neutral was achieved, whereupon implant removal was undertaken. Continued follow-up is then recommended on an annual basis, until maturity. Four patients experienced recurrent valgus following implant removal (2 episodes for one patient). Recurrent deformity was managed by re-implantation of the metaphyseal screw or plate.

## Results

**Fig. 2:**
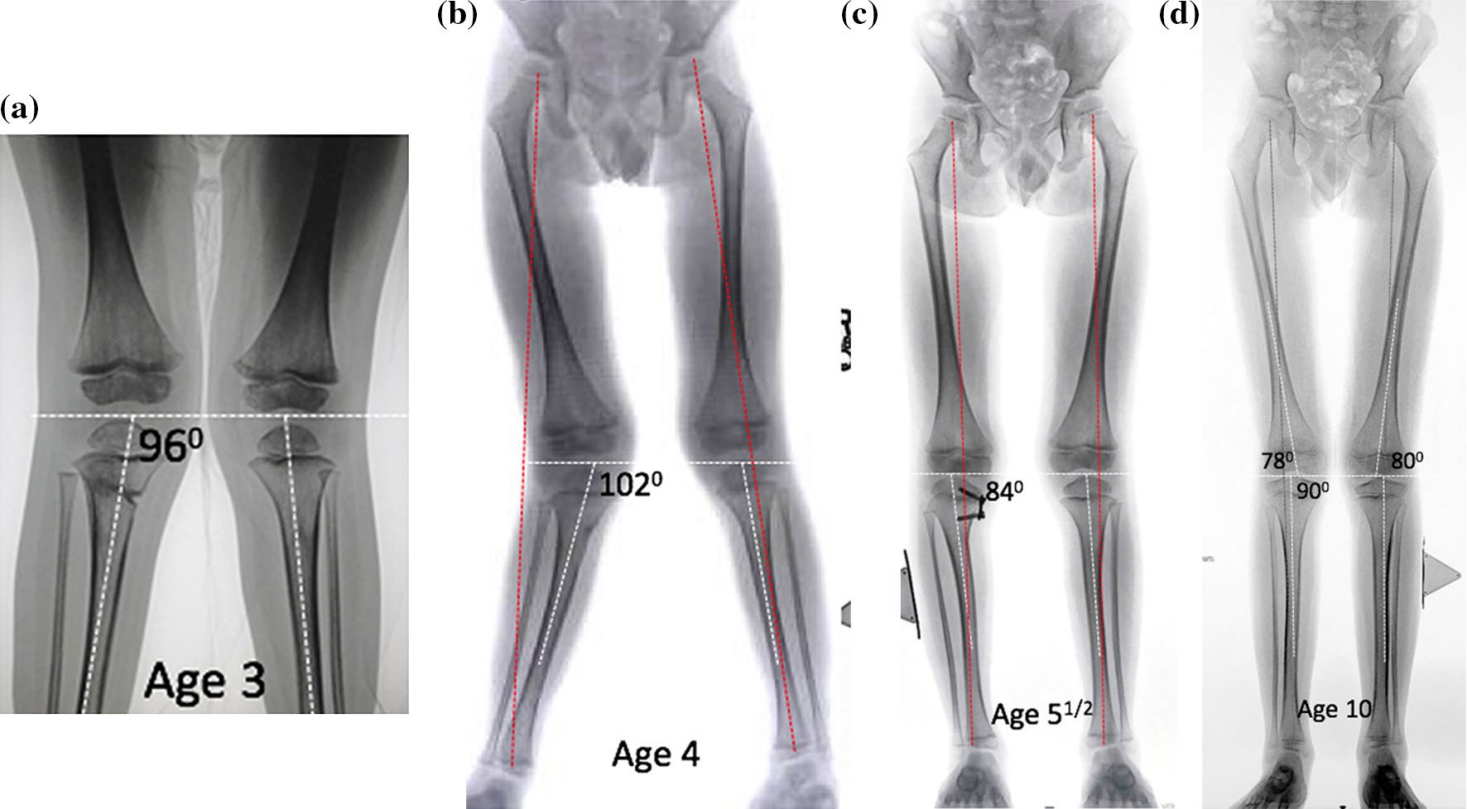
**a** Toddler who is 3 months status post-undisplaced proximal tibial fracture (intact fibula), treated in a cast. The proximal medial tibial angle PMTA is 96 degrees. **b** At follow-up, one year later, he has knee pain with activities. The PMTA has increased to 102 degrees, and the tibia is 9 mm longer. The mechanical axis is lateral zone +3. **c** 18 months following guided growth, his mechanical axis is intentionally overcorrected into medial zone -1 and the PMTA is 84 degrees. The implant was removed. **d** At age 10, he has mild and fairly symmetrical genu valgum (femoral). The mechanical axis is in lateral zone +1 bilaterally. His PMTA is 90 degrees (normal), and his tibial lengths are equal

The average age at follow-up was 10 years and 4 months. The average duration of hardware retention was 16 months. The mean follow-up, post-injury, was 7 years 3 months. It is important to note that physiologic valgus is common in children. Whether or not this is a predisposing risk factor for Cozen’s deformity is a matter of conjecture. Furthermore, genu valgum at follow-up may be due to femoral and/or tibial valgus (Fig. 2). Pre-operatively, all patients manifest a circumduction gait pattern due to the pathological genu valgum of the involved extremity, as judged by visual observation and consequent increase in intermalleolar distance, reaching as high as 12 centimetres. Clinical follow-up demonstrated a reduction in the intermalleolar distance and normalization of gait in all patients. For the ten patients who reached maturity, the limb length discrepancy averaged 0.05 centimetres (range 0.1–1.5 cm).

Serial radiographs demonstrated consistent and progressive improvement in limb alignment. The average medial proximal tibial angle (MPTA) was 107 degrees prior to guided growth, compared to a normal value of 87 degrees. This was measured using the anatomic axis of the tibia relative to a line drawn between the corners of the tibial metaphysis, given the varying degrees of ossification of the proximal tibial epiphysis. This improved to an average of 87.2 degrees after treatment compared to 86.7 degrees in the contralateral extremity. The lateral distal femoral angle (LDFA) averaged 86.4 degrees in the affected extremity at follow-up and 85.3 degrees in the contralateral extremity. This was measured using the anatomic femoral axis relative to a line drawn at the distal tips of the femoral condyles. In other words, we did not observe a concomitant, pathological femoral deformity. The average true limb length discrepancy was 4.9 mm pre-operatively and 4.7 mm at final follow-up.

The most useful measurement was the mechanical axis zone. This is refractory to magnification artefact, has minimal interobserver error, and is simpler to measure than MPTA. The MPTA also does not take into account potential deformities in the diaphysis and distal tibia. Prior to guided growth, the mechanical axis was located in lateral zone +2 or +3 in the involved extremity, compared with neutral to lateral zone +1 in the contralateral limb. Most patients (89%) had physiologic genu valgum on the unaffected side when looking at the zone of the mechanical axis. When measuring the mechanical axis deviation of the unaffected side, the average was 7 mm (range 2–14 mm). But when comparing the mechanical axis deviation of the affected to the unaffected side, there was always a significant difference (average 24 mm, range 11–44 mm).

Implant removal was undertaken when the mechanical axis of the affected extremity reached neutral to medial zone -1. Before treatment, the mechanical axis deviation averaged 30 mm lateral to the centre of the knee in the affected extremity compared to 6.6 mm in the uninvolved. Mechanical axis deviation at final follow-up was 10 mm on the affected side and 7 mm on the unaffected side. Out of 19 patients, 18 had a mechanical axis crossing through the middle of the knee or medial zone -1 at the knee at final follow-up.

One patient developed an early post-operative wound infection that required incision and drainage, plate removal, and antibiotic treatment. She underwent reinsertion of the plate 6 months later and realized uneventful correction. That was the only perioperative complication in this series. One of the older patients had a past surgical history of two failed attempts at tibial stapling commencing at age two (not recommended) using Blount staples. Each time, the staple loosened prematurely and was removed without full correction. The deformity persisted and was successfully managed with a plate at age 13 years and 4 months. One girl, who was 13 years and 6 months old at the time of tethering, did not achieve any correction because she had insufficient growth remaining at the time of implantation. Her mechanical axis remained in lateral zone +2, but she has not had sufficient complaints to warrant a corrective osteotomy. Two patients failed to return for the recommended follow-up, and consequently they experienced overcorrection into significant genu varum.

## Discussion

The aetiology of Cozen’s phenomena remains enigmatic. In his original article, Cozen postulated that lateral tethering of the IT band and weight bearing prior to fracture consolidation may have been the cause. Additional proposed aetiologies include interposition of periosteum into the fracture site [4, 16] and asymmetric growth stimulation of the physis [4, 9]. At presentation, we noted physiologic valgus (lateral zone +1) of the contralateral limb in most of our patients and believe that this may be a predisposing risk factor for the ensuing deformity. In all of our patients, we noted that the true length of the involved tibia exceeded that of the contralateral side by up to 1 cm. This would mitigate against the implication of lateral physeal damage or tethering as a factor, suggesting instead that the aetiology is an example “regional acceleratory growth phenomenon”, described by Frost [17]. Similar phenomena have been described in the distal tibia, distal femur, and distal humerus, leading to long bone deformity. Perhaps, the medial periosteal tether of the perichondrial ring is lost, resulting in unrestrained growth.

The initial and appropriate treatment of proximal tibia fractures usually consists of immobilization in a long leg cast, typically non-weight bearing, until fracture healing. Even for patients with no fracture displacement, the parents should be made aware of the possibility of post-traumatic medial tibial overgrowth. Ironically, and despite proper management, a “simple fracture” may have adverse consequences and an untoward outcome. In this series, none of the parents anticipated the insidious and progressive deformity to follow. If parents are not forewarned, they may understandably be unhappy with the treating physician. However, the timing of intervention for progressive Cozen’s deformity is open-ended. It is important to emphasize to the parents that, except for the unlikely prospect of encroaching skeletal maturity, intervention is not time sensitive. In order to rule out spontaneous resolution of valgus, their child may be seen biannually before committing to action.

Some practitioners still treat Cozen’s phenomena expectantly [9, 18]. However, Zionts et al. [9] reported that the mechanical axis remains at least 15 mm lateral to the knee centre in his series. It is unclear regarding the long-term consequences related to this, but they are likely undesirable. Mechanical axis lateralization can lead to abnormal circumduction gait, sometimes with consequent out-toeing, while altering joint reactive forces and provoking degenerative changes [19]. Although these effects take years to evolve, they may be averted by early intervention, taking advantage of the open physis to restore the mechanical axis and mitigate against limb length discrepancy. Given the choice between surgery and observation, all of the parents in this series have opted to proceed with guided growth rather than wait and hope for spontaneous resolution. We advise waiting at least a year post-fracture before offering intervention. Guided growth is not time sensitive, so the decision to intervene may be further postponed unless or until symptoms evolve. With the notable of the youngest patient in this series (initially stapled at age two), all of the patients in our series were at least 18 months post-fracture; none demonstrated spontaneous improvement and all were symptomatic.

We recently reported our success in preferentially managing patients with Blount’s disease by guided growth [20]. For those patients, we favoured early intervention, as soon as the mechanical axis reached medial zone -2. In the event of recurrent varus, we repeated the process as necessary, thereby avoiding osteotomy. The same logic applies to post-traumatic tibial valgus. The senior author previously reported the outcome of stapling for 12 children with progressive Cozen’s deformity [21]. Because of potential staple migration, we have since switched to using the tension band plate.

**Fig. 3:**
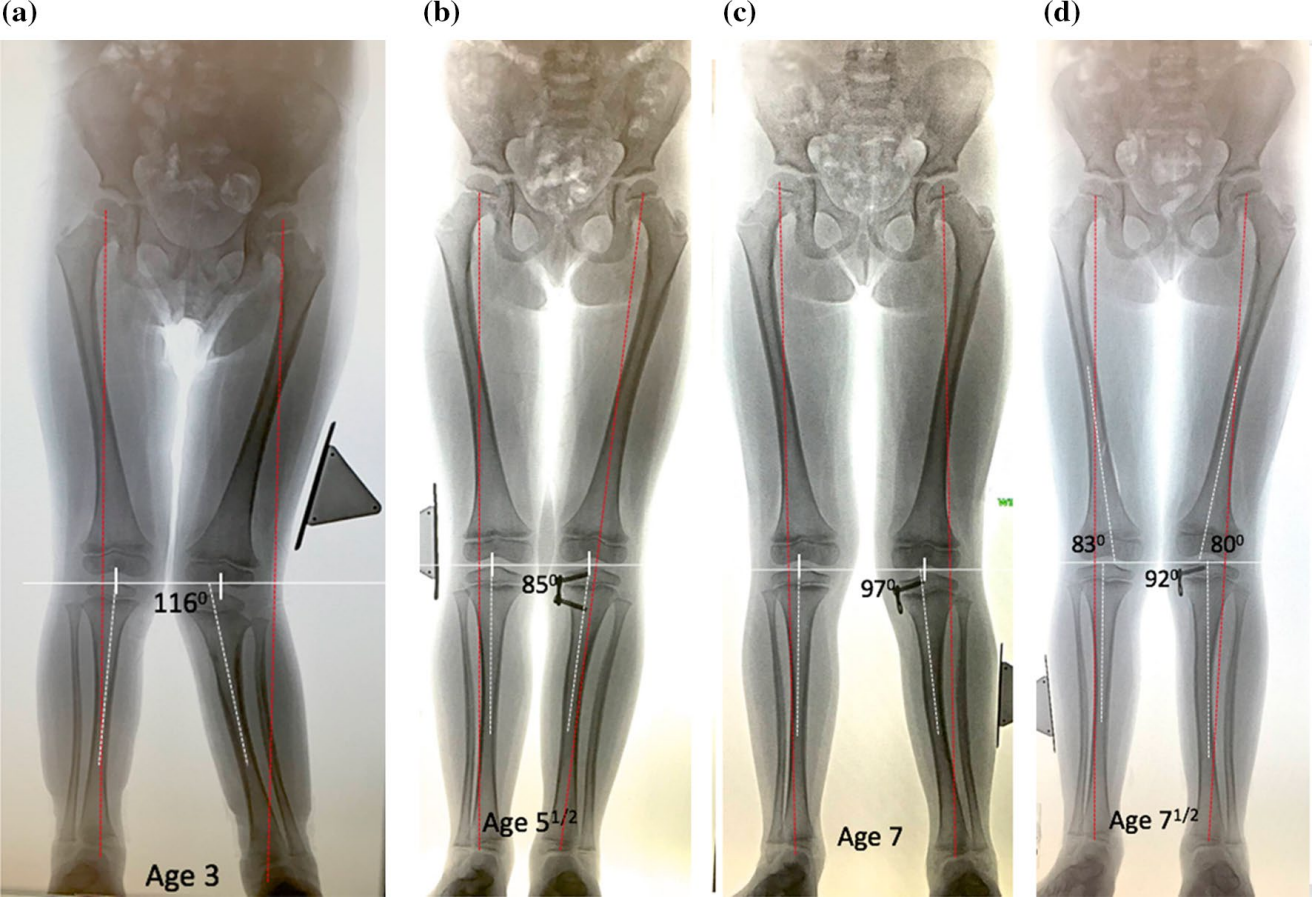
**a** This 3-year-old child presented 18 months following a proximal fracture of her left tibia. The mechanical axis was in lateral zone +3, and she had become symptomatic. **b** Two and a half years following guided growth of the tibia, her mechanical axis is neutral and the metaphyseal screw was removed. **c** At age seven, 18 months following screw removal, there is moderate recurrence of genu valgum and the mechanical axis is in lateral zone +2. **d** 6 months later, she remains asymptomatic, with slight improvement in tibial valgus. Note the distal femoral contribution to valgus. She will be monitored biannually, and the screw may be reinserted as indicated ± a femoral plate may be added for increasing valgus

Rebound growth can occur and is unpredictable [22]. We observed rebound valgus in four patients (one experienced this twice), three of whom corrected following re-implantation and the fourth just underwent his second metaphyseal screw reinsertion. For the skeletally immature patients, our current practice is to remove only the metaphyseal screw from the plate; this facilitates easy screw replacement should the deformity recur (Fig. 3). In this series, ten patients have completed their growth and are asymptomatic. We plan on following the remaining nine patients until skeletal maturity.

## Conclusion

Cozen’s phenomena of the tibia are manifested by medial tibial overgrowth following several causes including: fracture (most common), biopsy, bone graft harvest, traction pin insertion, and infection. By consensus, corrective osteotomy is contraindicated, due to rapid recurrence of the deformity. The philosophy of “expectant management” was based upon the misguided hope for spontaneous remodelling. It is now possible to safely correct the valgus deformity with minimal surgery. We recommend guided growth, tethering the proximal medial tibial physis with an extra-periosteal, non-locking plate, and two screws. This is a simple outpatient procedure with no post-operative immobilization or activity restriction. Patients need to be followed until they reach skeletal maturity, with repeat intervention, if necessary.
